# Aortic Stenosis Patients With Transcatheter Aortic Valve Replacement: Caution Recommended With Renal Failure During Hospitalization

**DOI:** 10.7759/cureus.9384

**Published:** 2020-07-25

**Authors:** Pawandeep Kaur, Temitope Ajibawo, Timiiye Yomi, Neev Patel, Mizba Baksh, Edmond Okotcha, Saurabh Kataria, Rikinkumar S Patel

**Affiliations:** 1 Medicine, Sri Guru Ram Das Institute of Medical Sciences and Research, Amritsar, IND; 2 Internal Medicine, Brookdale University Hospital Medical Center, New York City, USA; 3 Medicine, University of Benin School of Medicine, Benin City, NGA; 4 Medicine, Byramjee Jeejeebhoy Medical College, Ahmedabad, IND; 5 Internal Medicine, Dr. Nandamuri Taraka Rama Rao University of Health Sciences, Vijayawada, IND; 6 Medicine, Vinnytsia Pirogov National Medical University, Vinnytsia Oblast, UKR; 7 Neurology, University of Missouri, Columbia, USA; 8 Psychiatry, Griffin Memorial Hospital, Norman, USA

**Keywords:** renal failure, acute kidney injury, aortic stenosis, new generation tavr, tavr, mortality

## Abstract

Objective

Our study aimed to assess the risk of in-patient mortality due to renal failure and other comorbidities in aortic stenosis (AS) patients undergoing transcatheter aortic valve replacement (TAVR).

Methods

We conducted a cross-sectional study using a Nationwide Inpatient Sample (NIS, January 2010 to December 2014) from the United States and included 33,325 patients with a primary diagnosis of AS. Logistic regression was used to evaluate the odds ratio (OR) for in-hospital mortality in AS by comorbidities including renal failure.

Results

The prevalence of renal failure in AS patients is 29.2%, and a higher proportion were males (60.1%) and non-white (14.1%). Major loss of function (96.6%) and in-hospital mortality (5.1%) were also proportionally higher in prevalence. Female patients (OR 1.35, 95% CI 1.20-1.51) had higher odds of in-patient mortality in AS patients. Race was a non-significant predictor for mortality risk. Patients with comorbid coagulopathy (OR 2.02, 95% CI 1.79-2.27) and heart failure (OR 1.62, 95% CI 1.39-1.89) have increased mortality in AS inpatients. After controlling confounders, renal failure was significantly associated with increased in-hospital mortality (OR 1.43, 95% CI 1.28-1.61) in AS patients.

Conclusion

Renal failure was prevalent in AS patients and was an independent factor that increases the risk of in-hospital mortality by 43%. Due to worse outcomes, more studies are required to evaluate risk-benefit ratio and strategies to improve health-related quality of life in post-TAVR patients with renal failure, and optimally decrease inpatient mortality.

## Introduction

Aortic stenosis (AS) is a common valvular abnormality and is ranked as the third most common cardiovascular disease. In the general population, prevalence is around 0.4% and about 1.7% in the elderly population above 65 years of age [1]. A rise in prevalence is seen in AS with increasing age with about 4% of elderly aged above 85 years are affected by worsening aortic sclerosis and AS. Some of the factors associated with the progression of AS are older age, cigarette smoking, and comorbidities like hypertension, obesity, diabetes, dyslipidemia, chronic renal disease, atherosclerosis, and coronary artery disease (CAD). Other hemodynamic-related factors (left ventricular systolic dysfunction and/or low cardiac output), and aortic valve-related factors like a bicuspid aortic valve, degenerative AS, valve calcification and regurgitation also worsen the disease process [2].

Nearly 125,000 individuals in the United States began treatment for end-stage renal disease (ESRD) in 2016 and more than 726,000 (two in every 1,000 individuals) were on dialysis or were living with a kidney transplant. The incidence of ESRD is higher among men and African Americans (three times more likely than whites). The total expenditure on medicare beneficiaries with renal disease was about $100 billion in 2015 which comprised over $64 billion invested for all medicare beneficiaries with chronic kidney disease [3]. Most patients with ESRD have numerous comorbidities along with experiencing the effects of advanced AS. The prognosis of ESRD patients is affected by the presence of comorbidities like peripheral vascular disease, CAD, congestive heart failure (CHF), diabetes, and chronic pulmonary disease with nearly 50% one-year survival in elderly patients on dialysis [4].

Preoperative severe renal disease and also acute kidney injury (AKI) are major predictors for one-year mortality in transcatheter aortic valve replacement (TAVR) patients. Post-TAVR individuals with AKI had a mortality rate of 7.8% to 29% and two to eight times higher risk compared to patients who did not have AKI [5,6]. Individuals with ESRD and AKI have increased in-hospital mortality, increased occurrence of in-hospital complications, and poor outcomes following TAVR [7]. Individuals with advanced AS and ESRD undergoing TAVR have a remarkably elevated risk of mortality at 30 days and one-year and increased in-hospital mortality compared to non-dialysis patients [4].

In our study, we aim to discern the differences in demographics and comorbidities, and inpatient outcomes including the severity of illness, length of stay (LOS), total charges, and mortality between AS patients undergoing TAVR with versus without renal failure. Next is to evaluate the prevalence and risk of in-hospital mortality due to renal failure in post-TAVR patients.

## Materials and methods

Data source

A retrospective cross-sectional study was conducted using the National Inpatient Sample (NIS) database from January 2010 to December 2014. The NIS is the largest inpatient database from the healthcare cost and utilization project (HCUP) that includes patient records from about 4,411 hospitals and covers 45 states in the United States [8].

Inclusion criteria

We included adult inpatients ≥18 years of age with a principal discharge diagnosis of AS (N = 33,325) by identifying the primary diagnostic field in the NIS for the International Classification of Diseases, ninth revision (ICD-9) diagnosis codes for 395.0, 395.2, 396.0, 396.2, 424.1 or 746.3, and principal procedure of TAVR (ICD-9 procedure codes 350.5 or 350.6). The study population was grouped by comorbid renal failure (RF) by identifying the co-diagnoses fields for ICD-9 codes of 403.01, 403.11, 403.91, 404.02, 404.03, 404.12, 404.13, 404.92, 404.93, 585.3, 585.4, 585.5, 585.6, 585.9, 586, v42.0, v45.1, v45.11, v45.12, v56.0-v56.32, or v56.8.

Variables of interest

Demographic variables assessed in this study included age, sex, and race. To measure the differences in inpatient outcomes in AS patients undergoing TAVR by comorbid renal failure, the following variables were included: severity of illness, LOS, total charges, and in-hospital mortality. The severity of illness in the NIS is stratified by minor, moderate, major, and extreme loss of body functions. The in-hospital mortality is the number of inpatient deaths and is all-cause. The LOS is calculated by the number of nights the patient was hospitalized for AS and the total charges during hospitalization excluded professional fees or other non-covered charges during HCUP data processing [9].

Comorbidities are coexisting conditions to AS, and based on existing literature, we identified comorbidities including CHF, chronic pulmonary disease, and coagulopathy using ICD-9 diagnosis codes [9]​.

Statistical analyses

We used descriptive statistics and cross-tabulation to discern the differences in demographics and hospital outcomes, and comorbidities between AS inpatients with versus without RF. Independent sample t-test was used to measure the differences in continuous variables: LOS and total charges. We applied the discharge weight provided in the NIS to get a national representation of the study population [9]. We used a binomial logistic regression model to evaluate the odds ratio (OR) for in-hospital mortality by comorbidities including comorbid RF in AS inpatients with TAVR, and the model was adjusted for and age, sex, and race. A P-value <0.01 was used to determine the statistical significance of all the statistical analyses that were conducted using the Statistical Package for the Social Sciences (SPSS), version 26 (IBM Corp., Armonk, NY).

Ethical approval

To protect the privacy of patients and treatment information, the related identifiers were de-identified during the HCUP NIS data processing. We were not required to obtain institutional review board permission, as we utilized the publicly available de-identified data in our study [8].

## Results

We analyzed a total sample of 33,325 patients with AS, and 29.2% (N = 9,740) had comorbid RF. A higher proportion of AS patients with comorbid RF were male (60.1% vs. 47.5%) and non-white being black, Hispanic, or other (14.1% vs. 9.7%) compared to the non-RF cohort. Patients in the RF cohort had a higher prevalence of comorbidities compared to non-RF cohort (90.5% vs 60.2%), with a higher proportion of CHF (12.7% vs 11.2%) and coagulopathy (27.4% vs 21.3%). However, no statistically significant difference was seen in the prevalence of chronic pulmonary diseases between both cohorts. Major loss of function or severity of illness was seen in a higher proportion of AS patients in the RF cohort (96.6% vs 92.5%), and so was the In-hospital mortality (5.1% vs 3.6%) compared to the non-RF cohort. Statistically, there was a significant difference between the mean LOS and total charges as shown in Table 1.

**Table 1:** Differences in demographics and inpatient outcomes in aortic stenosis SD: standard deviation; USD: United States dollars

Variable	Renal failure (-)		Renal failure (+)		P-value
	N	%	N	%	
Total inpatients	23585		9740		-
Mean age (SD), in years	81.2 (8.28)		81.7 (7.55)		<0.001
Sex					
Male	10240	47.5	7085	60.1	<0.001
Female	11300	52.5	4700	39.9	
Race					
White	18185	90.3	9285	85.9	<0.001
Black	600	3.0	560	5.2	
Hispanic	515	2.6	370	3.4	
Other	835	4.1	595	5.5	
Comorbidities					
Chronic pulmonary disease	7190	33.4	4090	34.7	0.015
Congestive heart failure	2410	11.2	1500	12.7	<0.001
Coagulopathy	4590	21.3	3230	27.4	<0.001
Severity of illness					
Minor/moderate loss of function	1605	7.5	400	3.4	<0.001
Major loss of function	19935	92.5	11385	96.6	
Other hospital outcomes					
Mean length of stay (SD), in days	7.1 (6.14)		8.7 (7.54)		<0.001
Mean total charges (SD), in USD	207,765 (118,932)		223,568 (130,000)		<0.001
In-hospital mortality	780	3.6	605	5.1	<0.001

Female patients with AS have 1.35 times higher odds (95% CI 1.20-1.51) for in-hospital mortality compared to males. When compared to whites, no statistically significant association with inpatient mortality was seen in any other race. Among comorbidities, the association with in-patient mortality was seen with coagulopathy by two times (95% CI 1.79-2.27), and CHF by 1.62 times (95% CI 1.39-1.89). After controlling for demographic confounders and other comorbidities, the odds of in-hospital mortality was higher in patients with comorbid AF by 1.43 times (95% CI 1.28-1.61) as shown in Table 2.

**Table 2:** Risk of in-hospital mortality in aortic stenosis

Variables	Odds ratio	95% Confidence interval		P-value
		Lower	Upper	
Age	1.02	1.01	1.03	<0.001
Sex				
Male	Reference			
Female	1.35	1.20	1.51	<0.001
Race				
White	Reference			
Black	0.86	0.63	1.17	0.332
Hispanic	0.92	0.65	1.29	0.614
Other	1.08	0.83	1.39	0.574
Comorbidities				
No comorbidities	Reference			
Chronic pulmonary disease	1.09	0.98	1.24	0.123
Congestive heart failure	1.62	1.39	1.89	<0.001
Coagulopathy	2.02	1.79	2.27	<0.001
Renal failure	1.43	1.28	1.61	<0.001

## Discussion

Our study used the inpatient data from the US hospitals and found that the prevalence of RF in AS patients is 29.2%, and a higher proportion these patients were male (60.1%) and non-white being black, Hispanic, or other race (14.1%) compared to the non-RD cohort. Female patients had higher odds of in-hospital mortality in AS patients. Race was a non-significant predictor for mortality risk in our study inpatients. Our study showed that AS patients with comorbid coagulopathy and CHF had increased mortality. Major loss of function, severity of illness, and in-hospital mortality were seen in a higher proportion of AS inpatients with comorbid RF compared to the non-RF cohort.

AS is a common valvular abnormality with an approximate overall prevalence of 3% in the elderly over 75 years in the western world [10]. The incidence of AS rises with age, affecting up to 10% of the elderly by the eighth decade [11]. Risk factors associated with AS are older age, comorbidities such as diabetes and chronic renal failure, history of prior heart failure and left ventricular dysfunction, cardiac ischemic event, or revascularization [2]. When aortic valve sclerosis (thickening and calcification of the aortic valve) affects about 25% of elderly people then AS is prevalent in 2% to 9% of the elderly population over 65 years of age. A rise in prevalence was seen for both aortic sclerosis (48%) and AS (4%) in the elderly aged above 85 years [2].

We found that AS patients with comorbid RF had a higher prevalence of comorbidities (90.5%), with a higher proportion of coagulopathy (27.4%) and CHF (12.7%). One of the studies showed that most patients with ESRD have numerous comorbidities along with experiencing effects of advanced AS and prognosis of ESRD patients is highly affected by the presence of other comorbidities and their functional status. Comorbidities like peripheral vascular disease, coronary artery disease, CHF, and diabetes are associated with nearly 50% one-year survival in elderly patients on dialysis [4]. Another meta-analysis suggested that the occurrence of RF requiring hemodialysis following TAVR is related to baseline comorbidities instead of the amount of contrast used [12]. The presence of valvular abnormality is associated with higher in-hospital and 30-day mortality, and the clinical factors related to AS and aortic sclerosis are alike the risk factors for atherosclerosis, that is, old age, male gender, smoking, hypertension, and dyslipidemia [2].

As per our study, there was a statistically significant difference with higher mean LOS in AS inpatients with comorbid RF. According to a study, AKI accounts for up to a four-fold rise in one-year mortality, and individuals who developed AKI after TAVR had a mortality rate of 7.8% to 29% which was two to eight times higher as compared to patients who did not suffer from AKI. Also, AKI was associated with a higher LOS in AS patients who underwent TAVR, with a rise of about 2.5 times [6]. Renal dysfunction is one of the major factors responsible for adverse outcomes in individuals undergoing TAVR. Individuals with renal dysfunction usually have a high number of comorbidities and that may severely affect their life after TAVR.

The existence of renal dysfunction also affects the natural course of AS, probably via a mechanism that further contributes to calcium deposition in aortic leaflets, thus deteriorating AS. Advanced AS with reduced blood supply to major organs also contributes to the onset of complications, therefore enhancing the mortality risk after TAVR [12].

Deteriorating renal function manifested by estimated glomerular filtration rate (eGFR) decline was independently linked with a rise in all‐cause mortality in high‐risk individuals with AS. Initial stages of both chronic kidney disease and ESRD are associated with enhanced coronary as well as aortic and mitral valvular calcification and thus, a rise in parathyroid hormone, calcium-phosphate products, and excess 1,25 hydroxyvitamin D among other metabolic abnormalities. Valvular calcification is also associated with an enhanced rate of progression of AS and poor outcomes [13]. Increased morbidity and mortality related to TAVR are likely secondary to heavy calcification in the vasculature, aorta, and aortic annulus leading to bleeding and vascular complications [7].

A study showed that there are a variety of factors linked with AKI following TAVR such as high preoperative creatinine levels, the need for blood transfusion, peripheral vascular disease, and hypertension and thus, the short term mortality (<30 days) in patients who developed AKI after TAVR and was higher compared to those without AKI [14]. Baseline glomerular filtration rate is an independent predictor of acute kidney injury in patients undergoing TAVR which adversely affects 30-day and one-year mortality [15]. A study found that male sex, left ventricular ejection fraction <30%, atrial fibrillation, and advanced chronic kidney disease were independently associated with higher mortality in patients receiving TAVR in predialysis or dialysis individuals [16]. The presence of both severe chronic kidney disease and reduced LVEF and/or deteriorating functional status has a gradually progressive effect on mortality risk after TAVR [17]. Chronic kidney disease is predictive of enhanced 30-day and one-year all-cause mortality, AKI, dialysis requirement, adverse short and long-term outcomes following TAVR [18]. According to a multi-center study, individuals with advanced chronic kidney disease have remarkably high long-term mortality rates and also, various other factors such as age, male sex, lower ejection fraction are independently linked with high mortality rates following TAVR [16].

After controlling confounders, we found that comorbid RF was significantly associated with higher in-hospital mortality in AS inpatients in the post-TAVR period. According to a study, individuals with ESRD have increased in-hospital mortality, increased occurrence of in-hospital complications, and poor outcomes following TAVR. AKI is linked with increased in-hospital mortality in patients undergoing TAVR [7]. Individuals with advanced AS with ESRD who undergo TAVR have a remarkably elevated risk of mortality at 30 days and one year, and have increased in-hospital mortality and bleeding as compared to non-dialysis dependent individuals [4]. A decrease in eGFR in individuals with chronic kidney disease and AS is associated with an 18% higher risk of all‐cause mortality [13]. A meta-analysis demonstrated that females who underwent TAVR had greater long-term survival as compared to males but an increased short-term risk (<30 days) of major bleeding, vascular complications, and stroke [19].

There are a few limitations to our study. Administrative data from NIS were used to study inpatients as per the ICD-9 codes which may cause underreporting of comorbidities, including RF, thus affecting the statistical analyses. Since it is an observational cross-sectional study and all-cause in-hospital mortality was included, a causal relationship can’t be proved between mortality and comorbidities in AS. In order to strengthen our study, we utilized the NIS data covering hospitals from 44 states in the United States and the results have appropriate generalizability for the inpatient population. A logistic regression model adjusted for demographic confounders and comorbidities was used and thus, we evaluated the association of comorbidities including comorbid RF with in-hospital mortality in AS inpatients undergoing TAVR.

## Conclusions

Renal failure was prevalent in AS patients and was an independent factor that increases the risk of in-hospital mortality by 43%. Due to worse outcomes, more studies are required to evaluate the risk-benefit ratio and strategies to improve health-related quality of life in post-TAVR patients with RF, and optimally decrease inpatient mortality. Periodic renal function tests and appropriate management of chronic kidney disease need to be implemented at initial stages to improve health-related quality of life and decrease in-hospital mortality in AS patients.
